# Complex Evolutionary History of the Y Chromosome in Flies of the *Drosophila obscura* Species Group

**DOI:** 10.1093/gbe/evaa051

**Published:** 2020-03-16

**Authors:** Ryan Bracewell, Doris Bachtrog

**Affiliations:** Department of Integrative Biology, University of California, Berkeley

**Keywords:** Y chromosome, *Drosophila*, neo-sex chromosome, Y degeneration

## Abstract

The *Drosophila obscura* species group shows dramatic variation in karyotype, including transitions among sex chromosomes. Members of the *affinis* and *pseudoobscura* subgroups contain a neo-X chromosome (a fusion of the X with an autosome), and ancestral Y genes have become autosomal in species harboring the neo-X. Detailed analysis of species in the *pseudoobscura* subgroup revealed that ancestral Y genes became autosomal through a translocation to the small dot chromosome. Here, we show that the Y-dot translocation is restricted to the *pseudoobscura* subgroup, and translocation of ancestral Y genes in the *affinis* subgroup likely followed a different route. We find that most ancestral Y genes have translocated to unique autosomal or X-linked locations in different taxa of the *affinis* subgroup, and we propose a dynamic model of sex chromosome formation and turnover in the *obscura* species group. Our results suggest that Y genes can find unique paths to escape unfavorable genomic environments that form after sex chromosome–autosome fusions.

## Introduction

Sex chromosomes have formed independently many times from a pair of ordinary autosomes by acquiring a sex-determining gene (Bull 1983). In some species groups, such as many fish or reptiles, the proto-X and proto-Y keep recombining over most of their length and evolve little differentiation beyond the sex-determining gene (homomorphic sex chromosomes) (Kitano and Peichel 2012; Miura 2017). However, once the proto-sex chromosomes stop recombining over part or all of their length, they follow different evolutionary trajectories and differentiate genetically and morphologically (Charlesworth and Charlesworth 2000; Bachtrog 2013). Old Y chromosomes often are characterized by a loss of most of their original genes, an acquisition of male-specific genes, and an accumulation of repeats and heterochromatin. X chromosomes, in contrast, often evolve dosage compensation (Charlesworth and Charlesworth 2000).

Sex chromosome turnover can be frequent in some groups, especially if the X and Y show little differentiation (Vicoso 2019), but is thought to be rare for heteromorphic sex chromosomes (Bachtrog et al. 2014). The highly specialized gene content of old sex chromosomes (i.e., male-fertility genes on the Y) and chromosome-wide regulatory mechanisms (dosage compensation of the X, heterochromatin formation on the Y) is thought to make reversals of highly differentiated sex chromosomes into autosomes increasingly difficult (Bachtrog et al. 2014). Recent genomic studies, however, have uncovered turnover of heteromorphic sex chromosomes in multiple taxa. For example, the identity of the X chromosome was found to have changed multiple times across Diptera clades (Vicoso and Bachtrog 2015).

The evolutionary steps converting an autosome to a sex chromosome have been carefully studied at the molecular level in *Drosophila* using neo-sex chromosomes (Zhou and Bachtrog 2012, 2015). The fusion of autosomes to either or both of the ancestral sex chromosomes has repeatedly and independently created neo-sex chromosomes (i.e., an X-autosome fusion creates a neo-X, and a Y-autosome fusion creates a neo-Y). Neo-X chromosomes have evolved dosage compensation in multiple *Drosophila* species (Bone and Kuroda 1996; Marín et al. 1996; Ellison and Bachtrog 2013, 2019), whereas neo-Y chromosomes lose most of their genes, accumulate repetitive DNA, and become heterochromatic (Steinemann and Steinemann 1992; Zhou et al. 2013; Zhou and Bachtrog 2015; Mahajan et al. 2018).

**Fig. 1.**
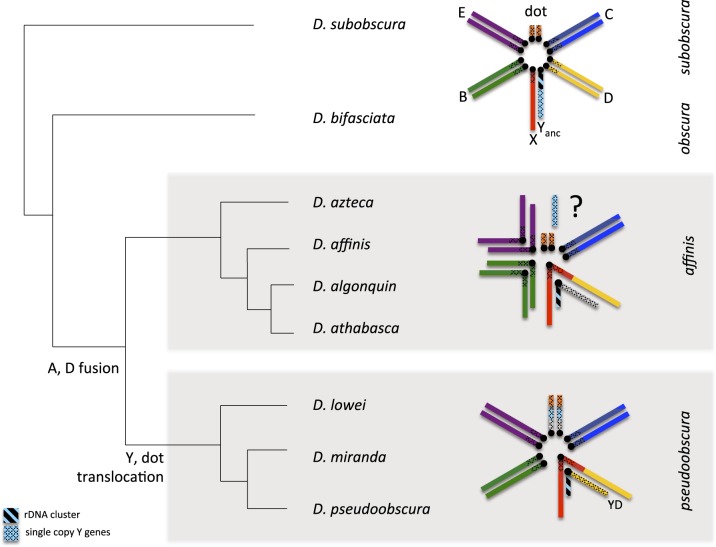
—Phylogenetic relationships and model of karyotype evolution of species in the *obscura* group (male karyotype). Only representative karyotypes that involve transitions of sex chromosomes are drawn (*Drosophila subobscura*, *D. athabasca*, *D. pseudoobscura*). The ancestral Y chromosome contains the repetitive rDNA cluster, and single-copy ancestral genes. Muller elements are color-coded; fragments of unknown origin are in gray.

Genomic comparisons, however, have also started to uncover examples in the reverse direction (that is, a sex chromosome reverting back to an autosome). In particular, the dot chromosome in *Drosophila*, a tiny autosome with strongly suppressed recombination, was ancestrally an X chromosome in flies (Vicoso and Bachtrog 2013). Indeed, multiple unusual features of this autosome can be better understood in light of its evolutionary history, such as the presence of a dosage-compensation machinery on the dot, or its peculiar expression patterns (Larsson et al. 2004; Riddle et al. 2009). Intriguingly, comparative analysis of Y-linked genes across *Drosophila* species also uncovered a Y to autosome reversion in members of the *obscura* species group (the *affinis* and *pseudoobscura* subgroups; see fig. 1).

In particular, five genes (*Ary*, *kl-2*, *kl-3*, *Ory*, and *Ppr-Y*) that were ancestrally present on the Y chromosome of *Drosophila* were found to all be autosomal in several members of the *affinis* and *pseudoobscura* subgroups (Carvalho and Clark 2005; Koerich et al. 2008). Detailed follow-up investigation and genomic analysis showed that the ancestral Y genes are incorporated in the dot chromosome in one piece in both *Drosophila* *pseudoobscura* and its relative *Drosophila* *miranda*, suggesting a chromosomal fusion or translocation creating this reversion (Larracuente et al. 2010; Chang and Larracuente 2017; Mahajan et al. 2018). Interestingly, members of the *affinis* and *pseudoobscura* subgroups also share a neo-X chromosome (Patterson and Stone 1952; Buzzati-Traverso and Scossiroli 1955). In an ancestor of these lineages, a former autosome (termed Muller element D) fused to the ancestral X chromosome (Muller element A) ∼15 Ma, and the neo-X has evolved the typical properties of an X (Sturgill et al. 2007; Ellison and Bachtrog 2013) (fig. 1). The fate of its former homolog (the Muller D element in males not fused to the X) was less clear. In some *Drosophila* species (such as *Drosophila* *americana*), X-autosome fusions result in two Y chromosomes (with the unfused chromosome forming a neo-Y), whereas in others (such as *Drosophila* *albomicans* and *Drosophila* *busckii*), the autosomes fuse to both the ancestral X and Y. Males in the *affinis* and *pseudoobscura* subgroups have a single Y chromosome, so it was initially assumed that an unfused neo-Y either completely degenerated, or that the neo-Y became incorporated into the ancestral Y and lost the majority of its genes (Patterson and Stone 1952; Buzzati-Traverso and Scossiroli 1955).

The discovery of a fusion or translocation between the ancestral Y and the dot chromosome led to an alternative hypothesis about the evolution of the Y in that species group (Carvalho and Clark 2005). Namely, it was suggested that the ancestral Y and neo-Y did not fuse after the X-autosome fusion, but that putative problems in meiosis that require pairing of multiple sex chromosomes were avoided by the fusion of the ancestral Y with the dot chromosome, and the current Y is a degenerate remnant of the neo-Y of this clade. Support for this notion came from genomic analysis of gene content of the Y chromosome in *D. pseudoobscura*, which was found to be enriched for genes from Muller element D (as would be expected if this chromosome formed from the neo-Y) (Mahajan and Bachtrog 2017).

Recent work involving more species, however, hints toward an even more complicated evolutionary history of the sex chromosomes in this clade (Dupim et al. 2018). In particular, although PCR analysis of the five ancestral Y genes confirms their presence in both males and females in most species of the *pseudoobscura* and *affinis* clade, some of those genes were found to be Y-linked in two species of the *affinis* subgroup: *Ary*, *kl-2*, and *Ory* could only be PCR-amplified from males in *Drosophila* *athabasca* and *Ary* and *kl-2* showed male-limited PCR-amplification in *Drosophila* *algonquin* (Dupim et al. 2018). This was interpreted as the “reappearance of Y-linkage” for some ancestral Y genes, or as the result of a Y duplication with a free copy of the Y chromosome remaining and one copy becoming incorporated into the dot chromosome followed by random inactivation of duplicate Y genes (Dupim et al. 2018). Here, we use genome analysis to reconstruct the evolutionary history of ancestral Y genes in the *obscura* group (fig. 1) by taking advantage of chromosome-level assemblies for nine different species (or semispecies). Contrary to current belief, our results suggest that the Y-dot fusion/translocation only happened in members of the *pseudoobscura* clade. Surprisingly, we find that ancestral Y genes independently moved away from the Y chromosome to different locations on the autosomes or the X in different species of the *affinis* subgroup. This suggests that Y-linkage of some ancestral Y genes in *D. athabasca* and *D. algonquin* is likely the ancestral configuration. We propose that the translocation of ancestral Y genes can best be understood as them escaping from the hostile genomic environment of a neo-Y chromosome, where they suffered the deleterious effects of genetic linkage to a large number of selective targets.

## Materials and Methods

Seven of the *Drosophila obscura* group genome assemblies (*D. athabasca* Eastern-A [EA] and Eastern-B [EB], *Drosophila* *lowei*, *D. miranda*, *D. pseudoobscura*, *Drosophila* *subobscura*, and *Drosophila* *bifasciata*) used in our analyses are described in detail in Mahajan et al. (2018) and Bracewell et al. (2019, 2020) and are available through GenBank (accessions: GCA_008121225.1, GCA_008121215.1, GCA_008121275.1, GCA_009664405.1, GCA_008121235.1, GCA_004329205.1, and GCA_003369915.2). For *Drosophila* *affinis*, we used a newly generated PacBio-based genome assembly kindly provided by Rob Unckless. For *Drosophila* *azteca*, we downloaded the most recent version from GenBank (accession: GCA_005876895.1) and additional details can be found at NCBI Bioproject PRJNA475270. To assign *D. azteca* contigs/scaffolds to Muller elements, we used D-Genies (Cabanettes and Klopp 2018) to perform whole-genome alignments with our other chromosome-level genome assemblies. During genome alignments and BLAST searches (below), we flagged contig VCKU01000055.1 as chimeric as it is a composite of sequences that map uniquely to different pericentromeric regions on all chromosomes in other assemblies. After identifying the Muller F from all assemblies, we generated alignments and dot plots using MUMmer (Kurtz et al. 2004) with NUCmer -mum -c 200 and mummerplot with the -filter option.

To find ancestral Y genes, we used the annotation file (gtf) and dot (Muller F) assembly from Chang and Larracuente (2017) along with gffread (https://github.com/gpertea/gffread) to generate transcripts of ancestral-Y genes for use in blastn searches with *obscura* group genome assemblies (above). We retained the longest transcript for these five genes (see supplementary fasta file, Supplementary Material online). To further confirm our blastn results, we downloaded all *Drosophila* *melanogaster* translations (r6.30) from FlyBase (flybase.org) and used tblastn to again search all *obscura* group assemblies. All blastn and tblastn searches had colocalized hits, except for *Ppr-Y*, which was only found using blastn searches with the *obscura* group transcript. Results from blastn searches can be found in supplementary table 2, Supplementary Material online. Only hits with ≥80% sequence identify were kept. BLAST searches of *D.* *azteca* for *kl-3*, *Ppr-Y*, and *Ory* also returned high-scoring hits to contig VCKU01000055.1 which are not shown due to it likely being an assembly artifact.

To estimate sequencing coverage over genes, we generated whole-genome sequencing data (Illumina) for an individual female of *D. azteca and D. affinis*, and males and females of *D. athabasca.* We extracted DNA using a Qiagen DNeasy kit following manufacturer’s recommendations. DNA libraries were prepared using the Illumina TruSeq Nano Prep kit and sequenced on a Hiseq 4000 with 100-bp PE reads. We downloaded *D. algonquin* Illumina data that have previously been deposited with the SRA (accession SRR5768634). To estimate coverage over genes, we used as a reference the longest *D. athabasaca* (EB) transcript for each gene from MAKER annotations (Bracewell et al. 2019) along with the *D. pseudobscura* transcripts for *kl-2*, *Ary*, and *Or*y. We then used BWA MEM (Li and Durbin 2009) to map all paired-end Illumina reads as single-end reads to these transcripts. Samtools (Li et al. 2009) was used to manipulate files and coverage over each transcript (gene) was estimated from the bam files using bedtools genomecov and groupBy (Quinlan and Hall 2010). To estimate coverage for *D. melanogaster*, we downloaded Illumina data from Wei et al. (2018) (SRA accessions: SRX3492597 and SRX3492598) and used methods outlined above but mapped reads to the longest *D. melanogaster* transcript for each gene (release 6.31, FlyBase).

We characterized gene expression of the five ancestral Y genes in *D. athabasca* by analyzing RNA-seq data from Bracewell et al. (2019). We first cleaned raw Illumina reads using SeqyClean (https://github.com/ibest/seqyclean) and then used the HISAT2 (Kim et al. 2015), Samtools (Li et al. 2009), and the StringTie pipeline (Pertea et al. 2015) to estimate FPKM (fragments per kilobase of transcript per million mapped reads) for all expressed transcripts. To create de novo transcriptomes and identify ancestral Y gene transcripts from gene expression data from the *subobscura* subgroup, *obscura* subgroup, and *affinis* subgroup, we analyzed male-specific RNA-seq data for *Drosophila* *guanche*, *D. obscura*, and *D. athabasca*. For *D. athabasca* and *D. obscura*, we used testis-specific data, either from above, or downloaded from the SRA (accessions DRX049912 and DRX049913). For *D. guanche*, we downloaded data generated from whole adult males (accessions: ERX2096111, ERX2096112, and ERX2096113). Raw reads were cleaned using SeqyClean and we constructed de novo transcriptome assemblies using SPAdes version 3.14 (Bankevich et al. 2012) and default settings. We then identified ancestral Y transcripts from each assembly using blastn. Ancestral Y transcripts were aligned using MAFFT version 7 (Katoh and Standley 2013).

Plots of Muller F assemblies and locations of ancestral Y insertions were created using KaryoploteR (Gel and Serra 2017). Genes shown with *D. melanogaster* gene names are the result from tblastn searches (above) and only top hits with ≥50% sequence identity were plotted. To estimate repeat density in *D. azteca*, we used Repeatmasker version 4.0.7 (Smith et al. 2013–2015) with the -no_is and -nolow flags and the Repbase *Drosophila* repeat library (downloaded March 22, 2016, from www.girinst.org). The proportion of repeat-masked bases (Ns) in nonoverlapping windows along the masked genome was determined using bedtools nuc.

## Results

### Y-Dot Translocation Is Only Present in the *pseudoobscura* Subgroup

**Fig. 2.**
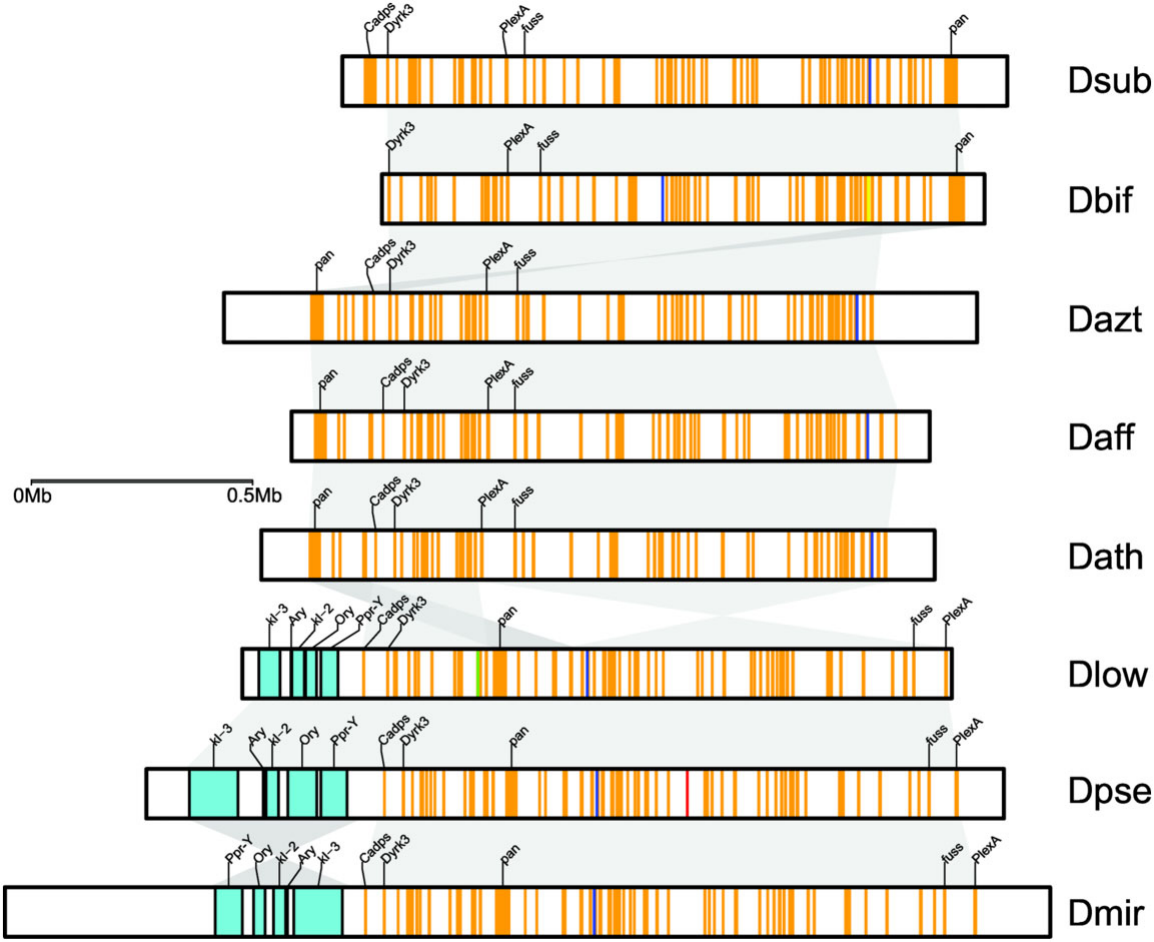
—Gene content of the dot chromosome in *obscura* group flies. Shown is the origin of dot genes (orange = Muller F; turquoise = ancestral Y; red = Muller A; green = Muller B; blue = Muller C; yellow = Muller D). Flies from the *pseudoobscura* subgroup all contain ancestral Y genes on the dot chromosome (turquoise), which are absent in other *obscura* group flies, including species from the *affinis* subgroup. The location of best BLAST hit is shown along with the inferred full-length coordinates for ancestral Y genes. Syntenic blocks (>100 kb) shown in gray. Select genes shown overtop each dot chromosome assembly (see supplementary fig. 1, Supplementary Material online, for all genes).

The *pseudoobscura* subgroup consists of five described species, and we recently completed chromosome-level genome sequences for three of them (Mahajan et al. 2018; Bracewell et al. 2019). For each of the three species (*D. lowei*, *D. miranda*, *D. pseudoobscura*), the dot chromosome was assembled in a single contig (fig. 2, table 1, and supplementary fig. 1, Supplementary Material online). Importantly, in each species, we detect the five ancestral Y genes assembled in a single genomic fragment, ranging from 180 to 357 kb. This fragment is in the same position at the end of each assembled chromosome (adjacent the genes *Cadps* and *Dyrk3*) although inverted in *D. miranda* relative to *D. lowei* and *D. pseudoobscura* (fig. 2 and supplementary fig. 2 and table 1, Supplementary Material online). Thus, our analysis supports that ancestral single-copy Y genes fused as a single segment to the dot chromosome in flies of the *pseudoobscura* subgroup and a lineage-specific inversion changed the linear order of the Y fragment in *D. miranda* (Larracuente et al. 2010; Chang and Larracuente 2017; Mahajan et al. 2018).

**Table 1:** Genome Assemblies of the Dot Chromosome (Muller Element F)

Species	Length (bp)	Contigs	Genes
Dsub	1,505,893	4	90
Dbif	1,364,133	1	90
Daff	1,445,299	1	NA
Dath EB	1,524,173	1	104
Dath EA	1,401,577	2	108
Dlow	1,606,711	1	108
Dmir	2,366,016	1	119
Dpse	1,941,385	1	101
Dazt	1,705,176	1	NA

Note.—NA: not available.

As expected, we find no ancestral Y genes on the dot chromosomes in *obscura* group species that lack the Muller A–D fusion (i.e., *D. subobscura* or *D. bifasciata*) and *Cadps* and/or *Dyrk3* are located at the end of the dot chromosome (fig. 2 and table 1). De novo transcriptome assemblies from males generated from a *subobscura* subgroup species (*D. guanche*) and an *obscura* subgroup species (*D. obscura*) recovered several transcripts with clear sequence similarity to *D. melanogaster* Y transcripts (supplementary text files, Supplementary Material online), indicating that ancestral Y genes are present in these lineages and located on the Y chromosome. These results are consistent with the hypothesis that the formation of the neo-sex chromosomes causes problems in meiosis, thus driving the fusion or translocation of the ancestral Y chromosome and the dot. Surprisingly, however, we also could not find any ancestral Y genes on the dot chromosome in our high-quality assemblies of two semispecies of *D. athabasca* (EA and EB), or in a chromosome-level assembly of *D. affinis* or *D. azteca* (fig. 2 and table 1). The lack of ancestral Y genes on the dot is unexpected, as the Y-dot translocation is thought to be shared by members of the *affinis* and *pseudoobscura* subgroups (Dupim et al. 2018). Previous analyses showed that none of the ancestral Y genes were male-limited in *D. affinis* and most other species in this subgroup (Dupim et al. 2018). Y-linkage of *Ary*, *kl-2*, and *Ory* in some lineages of the *affinis* group was interpreted as these genes either gaining Y-linkage secondarily, or as a Y duplication in an ancestor of the *affinis*/*pseudoobscura* group followed by random gene inactivation of duplicate Y genes on either the free Y chromosome or the Y copy on the dot (Dupim et al. 2018).

**Fig. 3.**
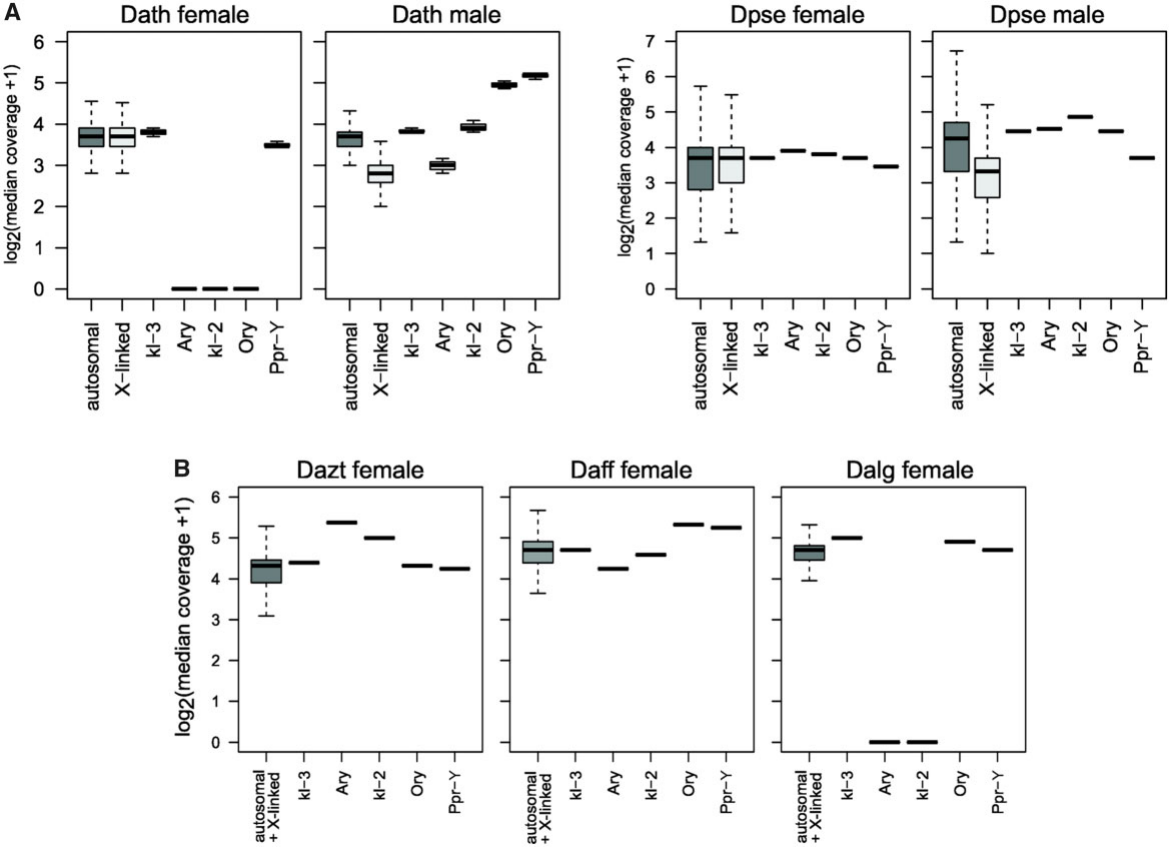
—Sex-linkage of ancestral Y genes in *affinis* group flies. (*A*) Shown is sequencing coverage of males and females for genes in *Drosophila athabasca* and *D. pseudoobscura*. (*B*) Shown is genomic coverage of genes for females in *D. azteca*, *D. affinis*, and *D. algonquin*. Outliers not shown for X-linked and autosomal genes.

Consistent with the PCR results (Dupim et al. 2018), we find all five ancestral Y genes in female Illumina libraries from *D. affinis* and *D. azteca* (fig. 3). Likewise, we detect *kl-3*, *Ory*, and *Ppr-Y* in reads from a female *D. algonquin* library but not *kl-2* or *Ary*. We find that *kl-3* and *Ppr-Y* are present in female *D. athabasca* but not *Ary*, *kl-2*, and *Ory* (fig. 3). Each of the ancestral Y genes, however, is clearly present in reads from a male genomic library of *D. athabasca*, implying that copies of these genes are found on the male-limited Y chromosome. Genomic read coverage suggests that some of the ancestral Y genes may be present in multiple copies. For example, median read coverage in male and female *D. athabasca* supports one autosomal copy of *kl-3*, one Y-linked copy of *Ary*, whereas increased male read coverage suggests two Y-linked copies of *kl-2*, and multiple Y-linked copies (or parts of) for *Ory* and *Ppr-Y* (fig. 3). Likewise, read-coverage analysis supports multiple (possibly partial) copies of *Ary* and *kl-2* in female *D. azteca*, and possibly multiple (partial) copies of *Ory* and *Ppr-Y* in female *D. affinis* (fig. 3). It is important to note, however, that detecting small changes in copy number (or gene fragments) using read coverage is challenging; when applying these methods to the reference strain of *D. melanogaster*, we consistently found lower than expected male coverage of ancestral Y genes (supplementary fig. 3, Supplementary Material online).

### Independent Incorporation of *kl-3* and *Ppr-Y* on Muller B of *D. athabasca*

**Fig. 4.**
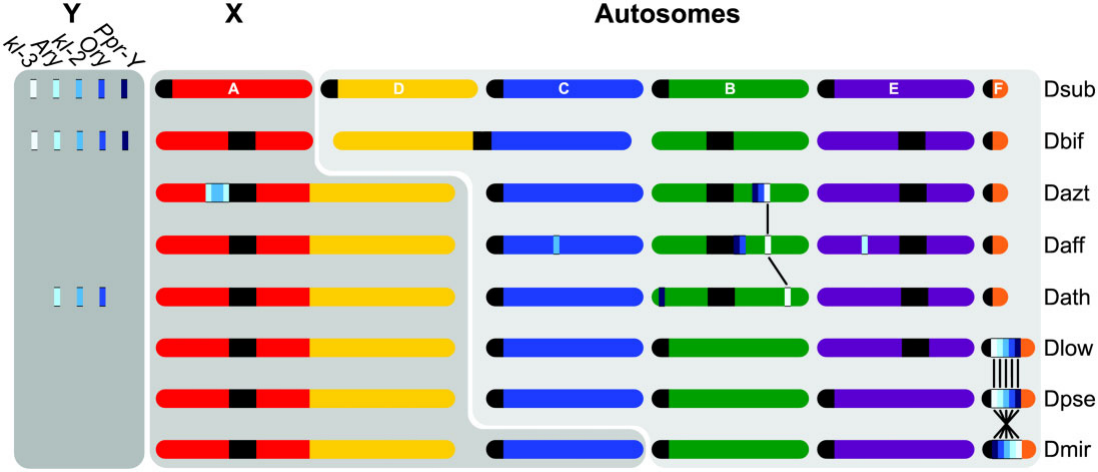
—Schematic representation of location of ancestral Y genes in *obscura* group flies. Shown is the approximate genomic location of the five Y_anc_ genes based on high-quality genome assemblies. The presence/absence of Y_anc_ genes on the Y chromosome is inferred from genomic coverage patterns (fig. 3). Muller elements are color-coded as in figure 1 and identified in *Drosophila subobscura*. Vertical lines connect genes found in homologous positions. Note that Muller C is a neo-X chromosome in some *D. athabasca* (similar to *D. miranda*), but for simplicity is not shown.

If not on the dot chromosome, where are ancestral Y genes found in *affinis* group flies? Consistent with our coverage analysis and PCR results (Dupim et al. 2018), we find *kl-3* and *Ppr-Y* to be contained in both of our female assemblies of EA and EB *D. athabasca*, but not *Ary*, *kl-2*, and *Ory*. Surprisingly, however, both *kl-3* and *Ppr-Y* are located on Muller B, in different chromosomal locations (fig. 4 and supplementary tables 1 and 2, Supplementary Material online). In particular, we find *Ppr-Y* on the short arm of Muller B (at ∼1.7 Mb), whereas *kl-3* is located on the long arm (at ∼37.7 Mb) in the EB assembly, and their locations are conserved in the EA semispecies. Thus, unlike the Y- to dot translocation in the *pseudoobscura* subgroup, we find that *kl-3* and *Ppr-Y* moved independently away from the Y chromosome to a different autosome in *D. athabasca*. We could not find *Ary*, *kl-2*, and *Ory* in our female assembly by BLAST (supplementary tables 1 and 2, Supplementary Material online), consistent with our Illumina read mapping and PCR results (Dupim et al. 2018).

Ancestral Y genes in *D. melanogaster* are expressed almost exclusively in testis (Gatti and Pimpinelli 1992). Testis expression patterns of ancestral Y genes have been conserved for *pseudoobscura* subgroup flies, where they moved as a single piece to the dot chromosome (Chang and Larracuente 2017; Mahajan et al. 2018). We used RNA-seq data from different male and female samples (male and female whole larvae, male and female adult and larvae heads; adult testis and ovaries) to investigate sex- and tissue-specific expression patterns of ancestral Y genes from both EA and EB *D. athabasca.* Consistent with these genes having important functions in *Drosophila* spermatogenesis, we find that they are all highly expressed in testis of *D. athabasca* (table 2). Thus, the genes that have stayed behind on the Y chromosome (*Ary*, *kl-2*, *Ory*) but also those that moved to an autosome (*Ppr-Y*, *kl-3*) have maintained their male-specific expression profile.

**Table 2:** Gene Expression of Ancestral Y Genes from Different Tissues and Sexes of Two *Drosophila athabasca* Semispecies (Eastern-A and Eastern-B)

		*kl-3*	*Ary*	*kl-2*	*Ory*	*Ppr-Y*
Eastern-B						
Male	Whole larvae	0.8	0	0	0	**1.3**
Male	Larval heads	0	0	0	0	0
Male	Testes	**67.2**	0	**11.3**	**39.54**	**73.8**
Male	Heads	0	0	0	0	0.7
Female	Whole larvae	0.4	0	0	0	0
Female	Larval heads	0	0	0	0	0
Female	Ovaries	0	0	0	0	**8.3**
Female	Adult heads	0	0	0	0	0
Eastern-A						
Male	Whole larvae	**2.5**	0	0.6	**2.3**	**3.6**
Male	Larval heads	0.0	0	0	0	0.7
Male	Testes	**81.3**	**10.2**	**12.1**	**44.5**	**292.2**
Male	Heads	**1.4**	0	0	0	0
Female	Whole larvae	0.6	0	0	0	0
Female	Larval heads	0	0	0	0	0
Female	Ovaries	0	0	0	0	0
Female	Adult heads	0	0	0	0	0

Note.—Values are in FPKM (fragments per kilobase of transcript per million mapped reads). Values of FPKM > 1 are in bold.

To conclude, our analysis confirms that *Ary*, *kl-2*, and *Ory* are still present in the male genome of *D. athabasca* but not in females, that is, these genes are located on the Y chromosome in this species. This is consistent with the PCR results of Dupim et al. (2018). However, they assumed that the Y-dot translocation was shared by *pseudoobscura*/*affinis* flies and therefore interpreted their PCR screen of *Ary*, *kl-2*, and *Ory* being only present in males as them becoming Y-linked secondarily or as the Y having been duplicated with a free copy and one incorporated into the dot followed by random gene loss. We find no evidence of ancestral Y genes on the dot, indicating that the Y-dot translocation is unique to flies in the *pseudoobscura* subgroup (but also, see Discussion for an alternative model). We show that *kl-3* and *Ppr-Y* independently became autosomal in *D. athabasca*, whereas *Ary*, *kl-2*, and *Ory* genes presumably never left the ancestral Y.

### Independent Y Gene Gain in *D. affinis* and *D. azteca*

In most species in the *affinis* subgroup (of which *D. athabasca* is a member), ancestral *Drosophila* Y genes are present in both sexes (fig. 3) (Carvalho and Clark 2005; Dupim et al. 2018). This was interpreted as a single Y-dot translocation moving all ancestral Y genes to an autosome (Larracuente et al. 2010; Dupim et al. 2018), but a lack of Y genes on the dot of *D. athabasca* and *D. affinis* argues against this scenario, and our results from *D. athabasca* suggest that ancestral Y genes may have been moved independently to autosomal locations in different species. To test this hypothesis, we analyzed high-quality genomes from *D. affinis*, a sister species to *D. athabasca* from which it diverged <3 Ma (Beckenbach et al. 1993), and *D. azteca* (which diverged <6 Ma; Beckenbach *et al.* 1993), two species for which all ancestral Y genes were found in both sexes. Indeed, we find copies for each ancestral Y gene in the female assembly of both species, but at strikingly diverse genomic locations (fig. 4 and supplementary tables 1 and 2, Supplementary Material online).

**Fig. 5.**
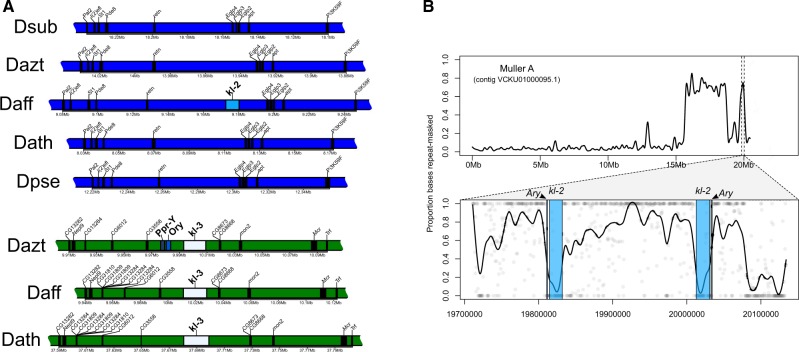
—Details of ancestral Y gene translocations. (*A*) Local alignments around *kl-2* indicate that this gene translocated to a region on Muller C (blue) in *Drosophila affinis*. Local alignments of the translocation of *kl-3* on Muller B (green) show it is in a homologous position in *D. azteca*, *D. affinis*, and *D. athabasca*. *Ppr-Y* and *Ory* appear absent from the region in *D. affinis* and *D. athabasca*. (*B*) *Ary*/*kl-2* are duplicated on XL (Muller A) of *D. azteca*, resembling palindromes found on the human Y chromosome. Shown above is a LOESS smoother fit to the proportion of bases repeat-masked in 500-bp windows. Below highlights the genomic interval harboring *Ary/kl-2.* Dots show individual 500-bp window estimates with a LOESS smoother fit to the genomic interval.

In particular, four of the five ancestral Y genes are found on different chromosomal locations in the *D. affinis* genome: *kl-2* is on Muller C (at 9.2 Mb), *kl-3* is on Muller B (10.0 Mb), *Ary* is on Muller E (9.8 Mb), and *Ory* and *Ppr-Y* appear to have translocated together onto Muller B (15.6 Mb). Comparisons of flanking regions suggest that the translocation of *kl-3* occurred in an ancestor of *D. affinis*/*D. athabasca*, as *kl-3* is surrounded by the same genes in both species (fig. 5*A*). *Ppr-Y*, on the other hand, is found on nonhomologous positions between *D. affinis*/*D. athabasca*, suggesting that this gene moved independently to Muller B in the two species. The *kl-2* translocation on Muller C in *D. affinis* appears to have only occurred in this species (fig. 5*A*).

Likewise, ancestral Y genes in *D. azteca* are located in different regions of the female genome assembly (fig. 4). *Ppr-Y*, *kl-3*, and *Ory* are found next to each other on Muller B (10.0 Mb), suggesting that they moved in one piece, and comparisons of flanking genes suggest that *kl-3* is located on a homologous position in *D. affinis* and *D. athabasca* (fig. 5*A*). Comparisons of this region in the *D. pseudoobscura* and *D. subobscura* genomes show that this Y gene translocation occurred at an *affinis* subgroup-specific inversion breakpoint (i.e., breakpoint relative to the *subobscura*/*pseudoobscura* subgroups), which limits our understanding of the size of the translocation. Our findings suggests that *kl-3* moved to Muller B in an ancestor of the *affinis* subgroup, and this initial translocation may have also included *Ppr-Y* and *Ory*, which were lost in the lineage leading to *D. athabasca*. An additional inversion may have moved *Ppr-Y* and *Ory* close to the pericentromere in *D. affinis* (but note that the long arm of Muller B appears completely syntenic between *D. affinis* and *D. azteca*, arguing against simple inversions; supplementary fig. 4, Supplementary Material online). *Ppr-Y* and *Ory* could also have moved secondarily onto the long arm of Muller B in *D. azteca* and independently in *D. affinis*, and *Ppr-Y* moved independently onto the short arm of Muller B in *D. athabasca.* Under either scenario, our results support a dynamic evolutionary history of ancestral Y gene movement in flies of the *affinis* subgroup. In *D. azteca*, we find that *Ary* and *kl-2* moved together to Muller A (the ancestral X chromosome), and both appear to be duplicated next to each other in opposite directions, with ∼180 kb of sequence in between them (fig. 5*B*). This insertion appears close to, or in, the pericentromere as the region has high repeat density and shows sequence similarity with pericentromeric regions in *D. athabasca* and *D. affinis* (fig. 5*B*). The sequence in between the *Ary*/*kl-2* duplication is almost entirely composed of repeats (75.1% repeat masked), and may thus be derived from the Y chromosome. The overall arrangement of *Ary* and *kl-2* resembles the palindrome structure of multicopy genes on the human Y chromosome (Rozen et al. 2003; Skaletsky et al. 2003), but it is unclear if this arrangement arose before or after these genes moved onto Muller A.

In summary, the absence of the Y-dot fusion, and a lack of conservation of location for most ancestral Y genes in the *affinis* subgroup indicates that genes moved away independently from the Y in this clade. Y genes in *D. melanogaster* can be gigantic, due to huge introns (Gatti and Pimpinelli 1992) and require unique gene expression programs (Fingerhut et al. 2019). Multiple independent translocations of ancestral Y genes suggest that the Y chromosome may have been smaller in *obscura* subgroup flies compared with *D. melanogaster*, which is consistent with karyotypic findings (Chang and Larracuente 2017).

## Discussion

The *obscura* species group of *Drosophila* provides a fascinating clade to study karyotype evolution (Bracewell et al. 2019), and it contains multiple sex chromosome transitions. Neo-sex chromosomes formed independently in different clades, including the fusion of the ancestral X with Muller D roughly 15 Ma, but also more recent fusions of Muller C with the Y chromosome in *D. miranda* and in some semispecies of *D. athabasca*, which allows us to reconstruct the events transforming an autosome into differentiated sex chromosomes. Intriguingly, however, we also observe the independent incorporation of ancestral Y genes in different species of *affinis* and *pseudoobscura* subgroup flies.

The ancestral Y of *Drosophila* contains both single-copy genes and the multicopy rDNA cluster (Hennig et al. 1975; Roy et al. 2005; Larracuente et al. 2010). FISH studies have shown that the rDNA cluster is present on both the X and the Y chromosome in multiple species of *obscura* flies, including members from the *obscura*, *affinis*, and *pseudoobscura* subgroups (Larracuente et al. 2010). This suggests that this is the ancestral configuration of the rDNA cluster, and its location on the Y was maintained even in species where single-copy Y genes translocated to the dot (*pseudoobscura* subgroup) or other chromosomes (*affinis* subgroup).

**Fig. 6.**
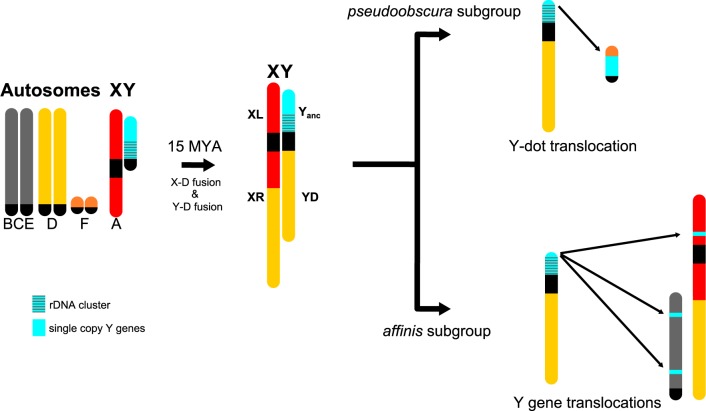
—Model of sex chromosome evolution in the *obscura* group. In an ancestor of the *affinis* and *pseudoobscura* subgroups, the ancestral X (Muller A) and Muller D fused ∼15 Ma. We hypothesize that the ancestral Y, which carries the rDNA cluster and single-copy Y_anc_ genes, also fused to Muller D, which would explain Y-linkage of the rDNA cluster in all species, and Y-linkage of Y_anc_ genes in several species. In the *pseudoobscura* subgroup, single-copy Y_anc_ genes translocated in one fragment to the dot chromosome, leaving behind (fragments of) Y_anc_ genes on the Y chromosome. In the *affinis* group, Y_anc_ genes moved independently to different autosomal and X-linked locations in different clades/species.

Although we cannot reconstruct the early events of sex chromosome evolution in the *obscura* group with certainty, we propose the following model that accounts for the genomic location of ancestral and newly formed sex-linked genes (fig. 6). In an ancestor of the *affinis*/*pseudoobscura* subgroups, the ancestral X fused to Muller D, and formed the second arm of the X chromosome found in all species belonging to these two subgroups. Such a fusion leaves the unfused Muller D, and the ancestral Y chromosome, and their fate has been less clear. Given Y-linkage of rDNA genes in species from all groups in *obscura* flies, this suggests that the rDNA cluster was ancestrally on the Y, and all species have incorporated at least part of the ancestral Y into their current Y (Larracuente et al. 2010). Additionally, some species in the *affinis* subgroup (*D. athabasca*, *D. algonquin*) have maintained ancestral single-copy Y genes on their current Y (see above; Dupim et al. 2018). Furthermore, an overabundance of Muller D genes was found on the current Y chromosome of *D. pseudoobscura* and *D. miranda* (Carvalho and Clark 2005; Mahajan and Bachtrog 2017; Mahajan et al. 2018), suggesting that Muller D (or part of it) also became incorporated into the Y of *pseudoobscura* subgroup flies. Thus, the simplest explanation for the current gene content of the Y in species with the X–D fusion is that Muller D also fused to the ancestral Y. Indeed, it is possible that the Y–D fusion actually preceded the X–D fusion, mimicking the current Y-autosome fusions found in *D. miranda* and *D. athabasca*, which would leave males with two unlinked X chromosomes. The fusion between either the X or the Y chromosome and Muller D would generate a trivalent in males (i.e., an X–D fusion creates two Y chromosomes in males, whereas a Y–D fusion would create two X’s in males that need to pair with one Y) and create problems in meiosis, resulting in higher rates of aneuploidy. This could rapidly select for a second fusion of Muller D with the unfused sex chromosome, as was experimentally demonstrated in a hybrid population of *D. albomicans* (a species that contains both a X-autosome and a Y-autosome fusions) and its sister species *D. nasuta* that lacks neo-sex chromosomes (Yu et al. 1999). If Muller D fused with both the ancestral X and Y, this should alleviate problems associated with segregating a trivalent. Ancestral Y genes then secondarily translocated to autosomal or X-linked locations, either as a single unit to the dot chromosome in an ancestor of the *pseudoobscura* subgroup, or individually to different chromosomal locations in species of the *affinis* subgroup (fig. 6). However, other more complicated scenarios are possible, including the Y-dot translocation happening in an ancestor of *affinis*/*pseudoobscura* flies, followed by a loss of all ancestral Y genes from the dot in *affinis* group species (see supplementary fig. 5, Supplementary Material online).

What might drive the relocation of ancestral Y genes? Becoming linked to a gene-rich chromosome will present a novel challenge for genes with important functions in spermatogenesis that have managed to survive for millions of years on a nonrecombining Y chromosome. In particular, evolutionary models to explain the degeneration of a Y are based on interference among selected mutations on a nonrecombining chromosome (Charlesworth 1978; Rice 1987). Theory and computer simulations have shown that the magnitude of selection interference, and thus the rate of degeneration, depends on the number of functional genes present on the Y chromosome (Bachtrog 2008). Gene loss is highest on a gene rich Y chromosome, but declines rapidly as active genes are lost (Bachtrog 2008). Although old, degenerate Y chromosomes may provide safe havens for important male-specific genes, and ancestral Y genes will suffer the deleterious effects of genetic linkage to more selective targets when fused to an autosome containing thousands of functional genes. Their translocation may thus be driven to avoid mutation accumulation and degeneration on the neo-Y where purifying selection is highly impaired. This resembles the fate of a Y gene (*kl-5*) in the *testacea* group species of *Drosophila* that duplicated to the dot chromosome (Dyer et al. 2011). The dot, like the Y chromosome, lacks recombination but contains about seven times more genes. It was shown that slightly deleterious mutations have accumulated in the dot-linked copy of *kl-5* faster than in the Y-linked copy (Dyer et al. 2011), consistent with the copy on the dot suffering the deleterious effects of genetic linkage to more selective targets compared with the Y chromosome.

Thus, our findings suggest a turbulent history of Y genes in the *obscura* group. After being protected from the accumulation of deleterious mutations on the gene-poor ancestral Y for millions of years, linkage to Muller D would have caused massive selective interference and degeneration of these genes. Y genes in the *pseudoobscura* subgroup escaped to a suboptimal genomic environment on the dot chromosome, whereas ancestral Y genes in the *affinis* subgroup began to duplicate or translocate to other autosomal locations. Therefore, a highly degenerate Y chromosome may not be as inhospitable as commonly assumed and may instead be a safe haven for male-beneficial genes.

A noticeable commonality between several of the ancestral Y gene translocations is that their autosomal copies are often found near heterochromatin. Ancestral Y genes fused to the heterochromatic dot chromosome in the *pseudoobscura* subgroup, *Ary*/*kl-2* are adjacent the pericentromere on Muller A in *D. azteca*, and *Ory*/*Ppr-Y* are near the pericentromere on Muller B in *D. affinis* (fig. 4). In addition, we found fragments of Y-linked genes in the pericentromeres of several other species and a small fragment of *Ory* even exists in a unique repetitive location on the end of the dot in *D. affinis* (supplementary table 2, Supplementary Material online). This suggests that ancestral Y genes may have an affinity for heterochromatin, and nonallelic homologous recombination between the repeat-rich Y chromosome and repetitive autosomal regions could facilitate movement of ancestral Y genes. Additionally, heterochromatin may be a preferential location for ancestral Y genes, as their regulatory machinery has evolved in a heterochromatic environment on the ancestral Y. 

## Acknowledgments 

We thank Rob Unckless for providing the *D. affinis* assembly. This work was supported by National Institutes of Health grants (NIH grants nos. R01GM076007, R01GM101255, and R01GM093182) to D.B.

## Supplementary Material

evaa051_Supplementary_Data

## Notes

**Data deposition:** All sequence data generated for this study have been deposited at the NCBI SRA under the NCBI BioProject accession PRJNA589338.
