# Supplementary material for: Complex Evolutionary History of the Y Chromosome in Flies of the Drosophila obscura Species Group
Source: Genome Biol Evol. 2020 Mar 16;12(5):494–505. doi: 10.1093/gbe/evaa051 (PMC7199386; doi:10.1093/gbe/evaa051)
Supplement: evaa051_Supplementary_Data [file evaa051_supplementary_data.zip › Legends supplementary materials.docx]

**Legends supplementary materials**

**Supplemental Figure 1.** Details of dot chromosomes (Muller F) from eight species in the *Drosophila obscura* group. *Drosophila melanogaster* (Dmel) gene names shown. Genes are color-coded based on their location in Dmel (orange = Muller F; turquoise = ancestral Y; red = Muller A; green = Muller B; blue = Muller C; yellow = Muller D).

**Supplemental Figure 2.** Dot plot comparisons (MUMmer) of the dot chromosome (Muller F) between species. Dot chromosome depictions on either axis are from Figure 2. A) Comparisons of the *Drosophila subobscura* dot (X axis) to *D. bifasciata*, *D. affinis*, *D. athabasca* (EB), *D. lowei*, *D. miranda* and *D. pseudoobscura*. B) All pairwise comparisons of *pseudoobscura* group species with the Y-dot fusion.

**Supplemental Figure 3.** Shown is sequencing coverage of males and females for ancestral Y genes in *D. melanogaster*.

**Supplemental Figure 4.** Dot plot comparisons (MUMmer) of Muller B between *D. affinis* and *D. azteca.*

**Supplemental Figure 5.** Models of Y-dot translocation in *affinis* flies*.*  Shown are two inferred inversions on the dot chromosome within the *subobscura* group based on the location of homologous genes. In model 1, the Y-dot translocation only happened in the *pseudoobscura* subgroup. In model 2, the Y-dot translocation occurred in an ancestor of the *affinis*/*pseudoobscura* group, but was lost secondarily in *affinis* flies. The presence of highly repetitive sequences on the dot and Y chromosome, and in particular near the pericentromere, complicates distinguishing between these scenarios.

**Supplemental Table 1.** Genome coordinates of five ancestral Y genes (*Ppr-y*, *Ory*, *Ary*, *kl-2*, *kl-3*) in published *Drosophila obscura* group genome assemblies. NA = not applicable.

**Supplemental Table 2.** Results of BLASTN searches with the longest transcript from ancestral Y genes on the dot (Muller F) chromosome of *D. pseudoobscura.* Results are in BLASTN output format 6 and separated by species. Each spreadsheet tab corresponds to a different ancestral Y gene.
