# Supplementary figures and images for: Complex Evolutionary History of the Y Chromosome in Flies of the Drosophila obscura Species Group
Source: Genome Biol Evol. 2020 Mar 16;12(5):494–505. doi: 10.1093/gbe/evaa051 (PMC7199386; doi:10.1093/gbe/evaa051)

A

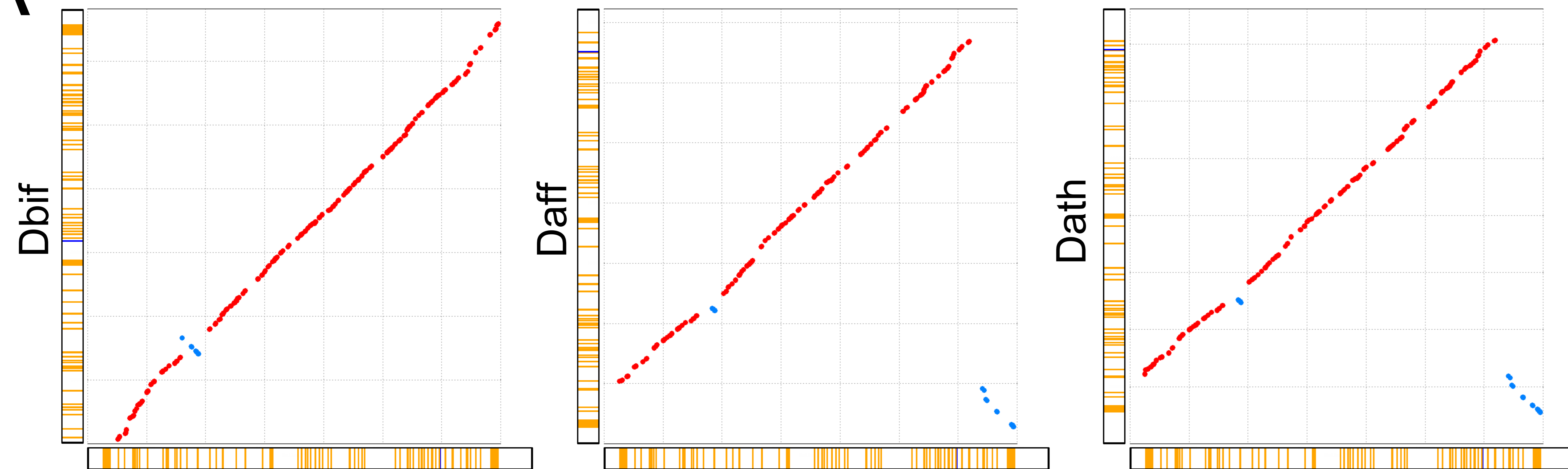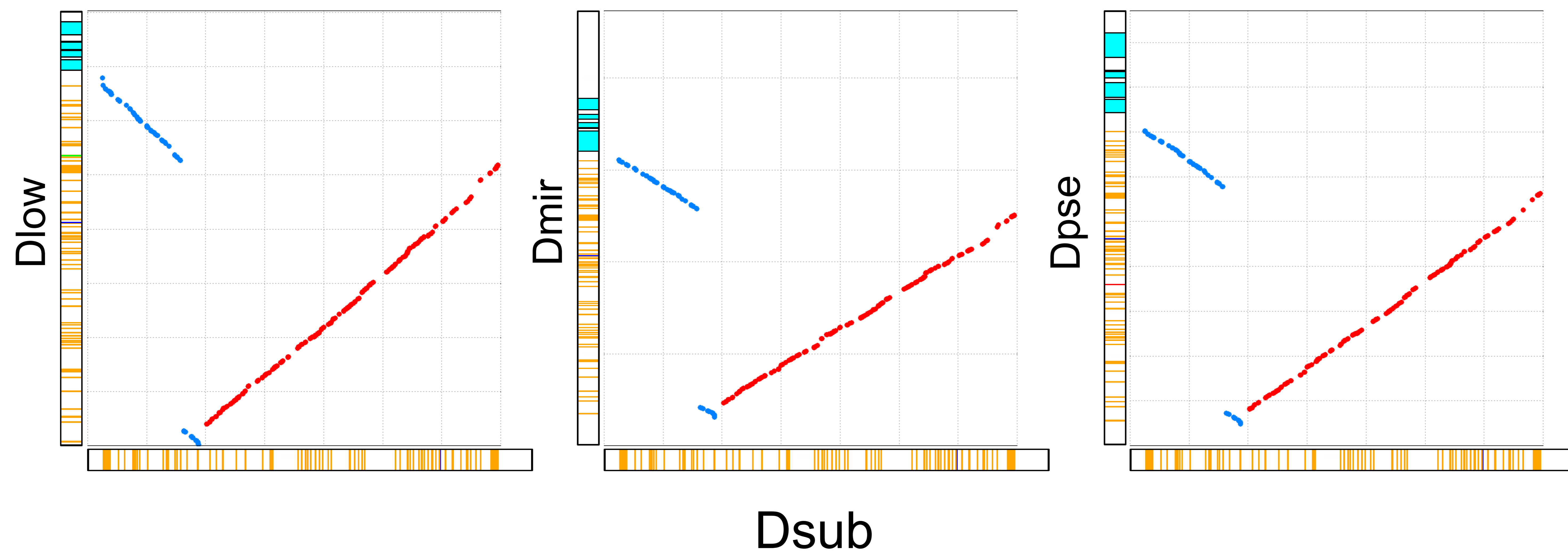

B

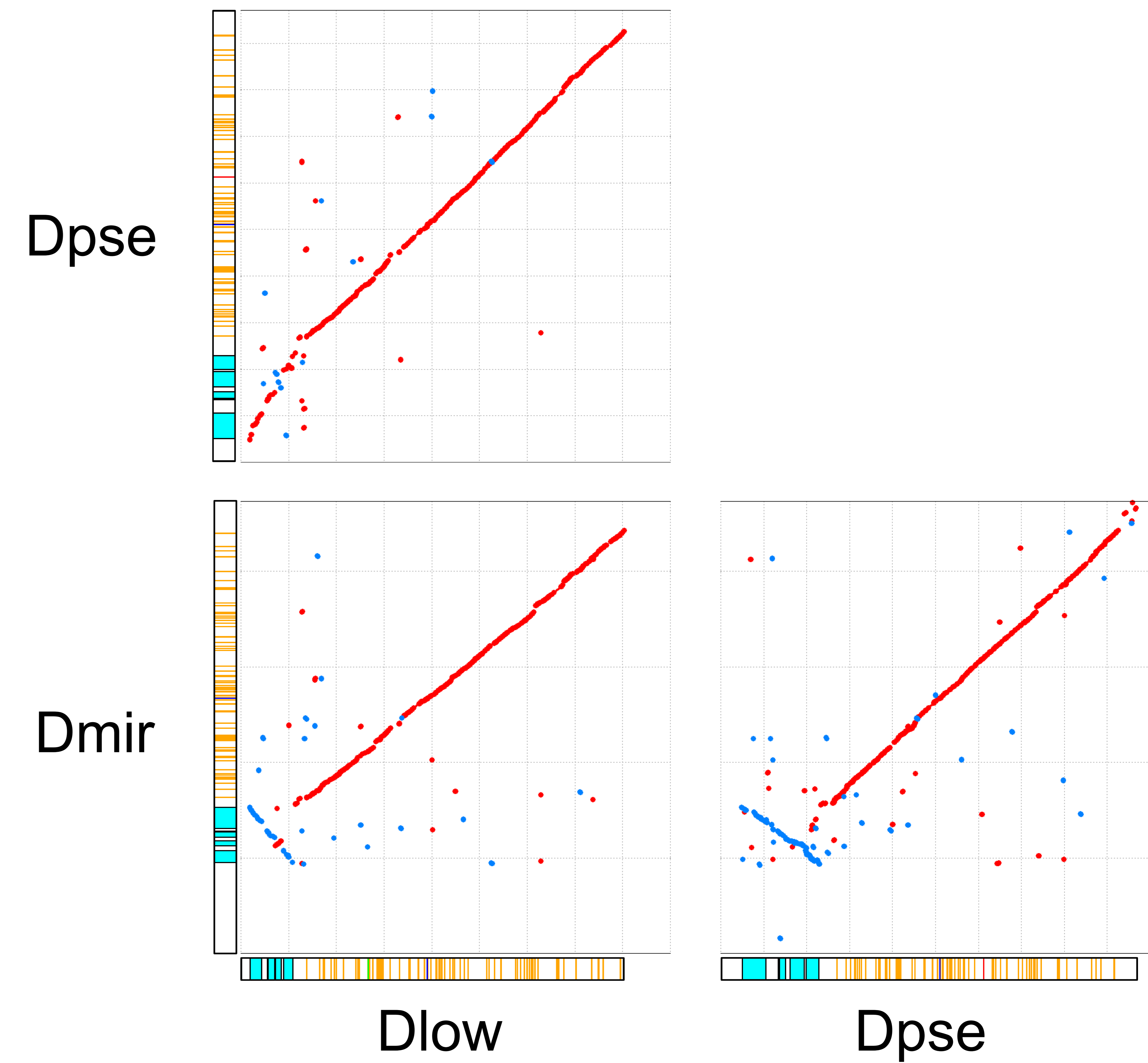

Supplement: evaa051_Supplementary_Data [file evaa051_supplementary_data.zip › Supplemental_Figure_2.pdf]

Dmel female

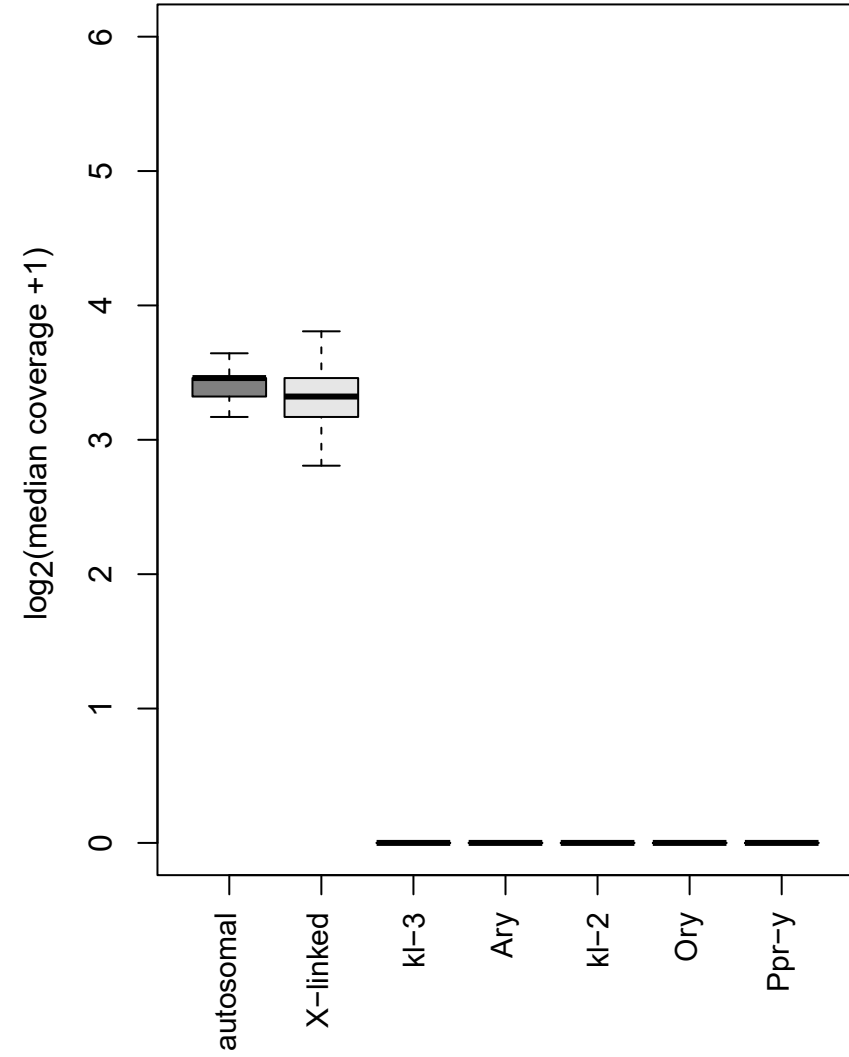

Dmel male

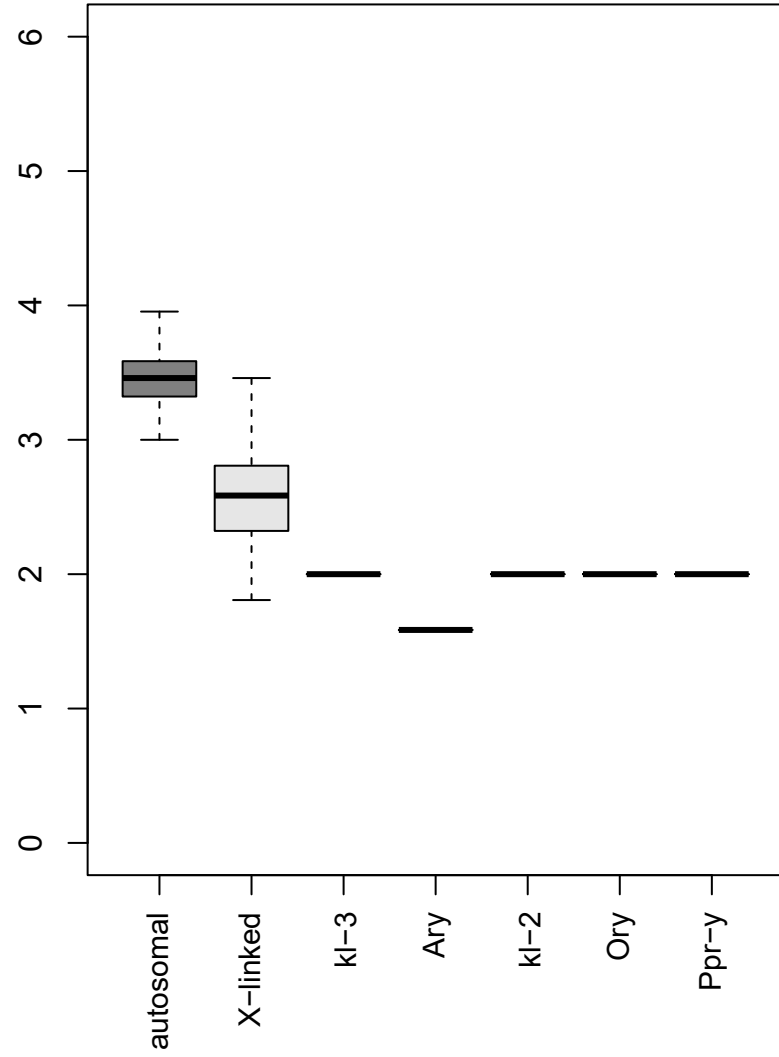

Supplement: evaa051_Supplementary_Data [file evaa051_supplementary_data.zip › Supplemental_Figure_3.pdf]

**D. affinis**

Chr4.group3\_MullerB

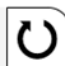

**D. azteca**

VC KU01000091.1

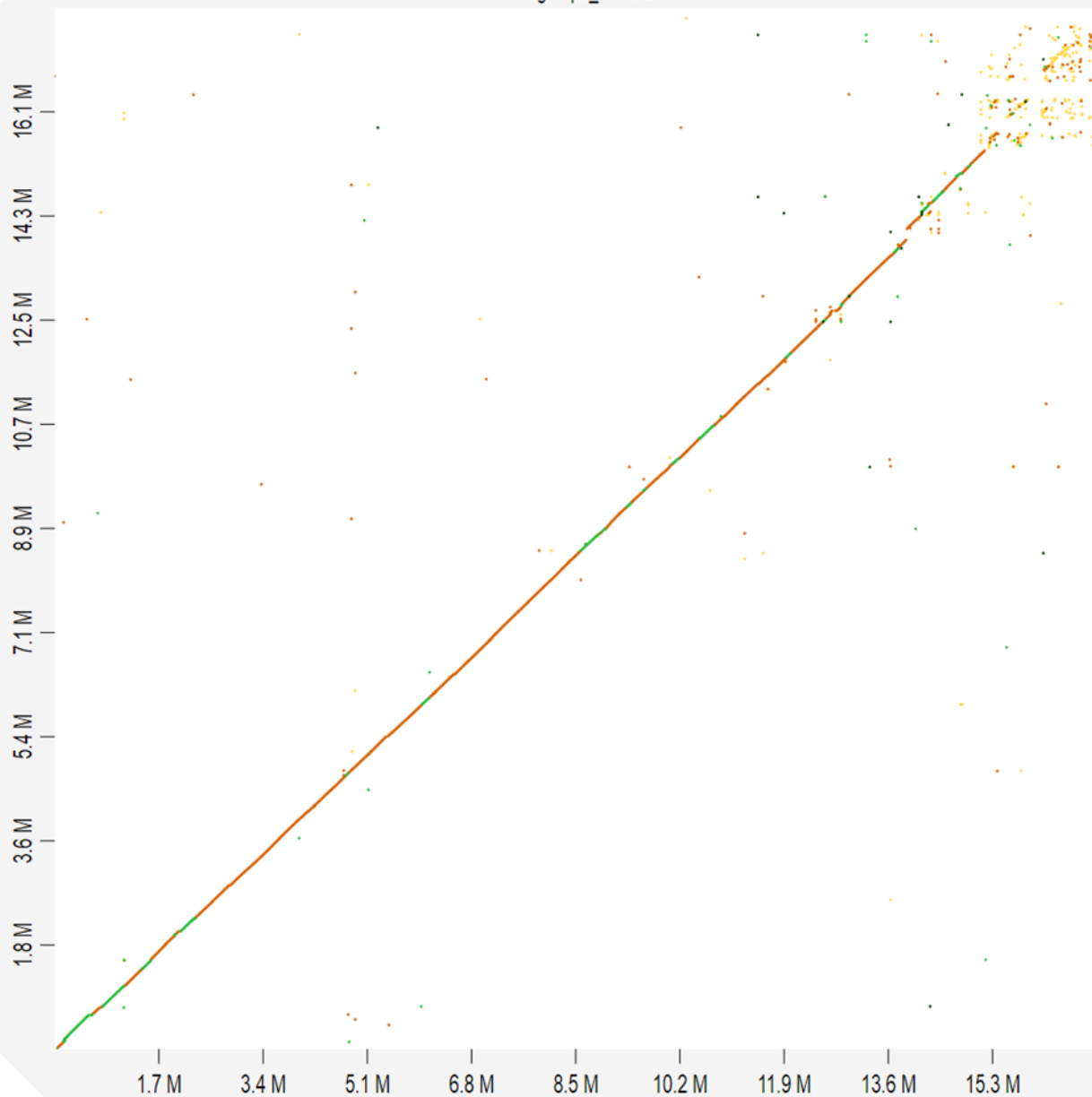

Supplement: evaa051_Supplementary_Data [file evaa051_supplementary_data.zip › Supplemental_Figure_4.pdf]

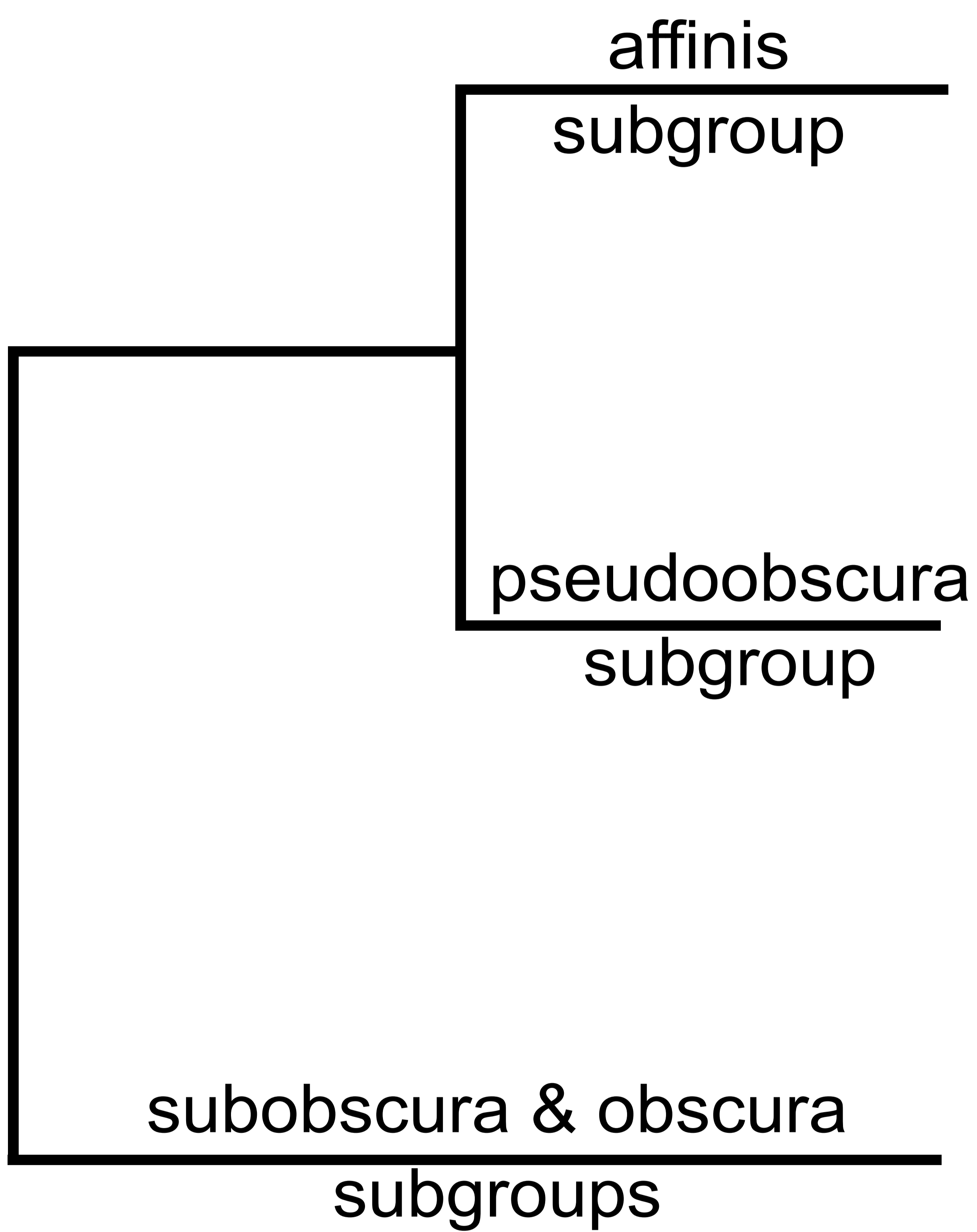

## Model 1

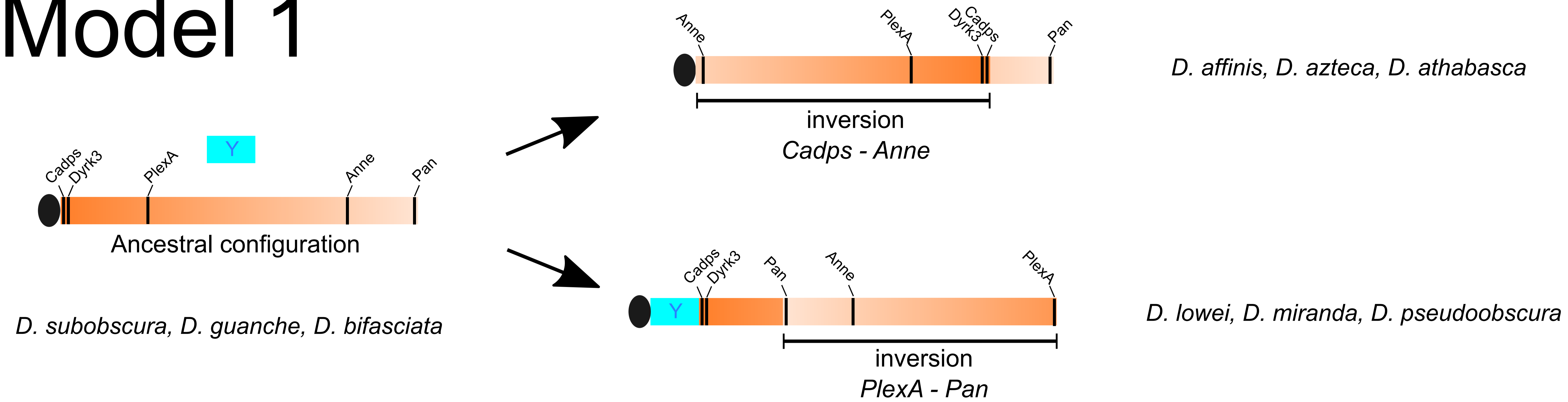

## Model 2

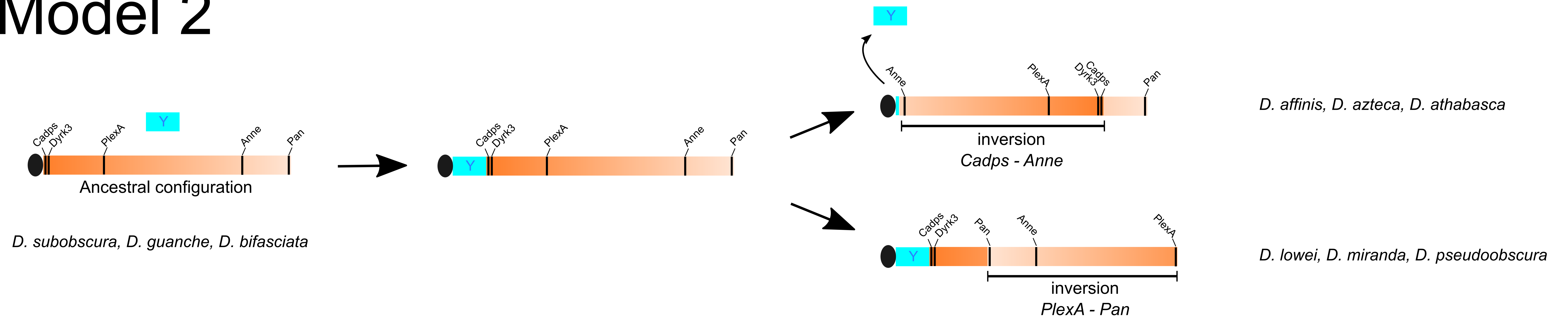

Supplement: evaa051_Supplementary_Data [file evaa051_supplementary_data.zip › Supplemental_Figure_5.pdf]
