# Supplementary material for: Complex Evolutionary History of the Y Chromosome in Flies of the Drosophila obscura Species Group
Source: Genome Biol Evol. 2020 Mar 16;12(5):494–505. doi: 10.1093/gbe/evaa051 (PMC7199386; doi:10.1093/gbe/evaa051)
Supplement: evaa051_Supplementary_Data [file Supplemental_Figure_1.pdf › Supplemental_Figure_1.pdf]

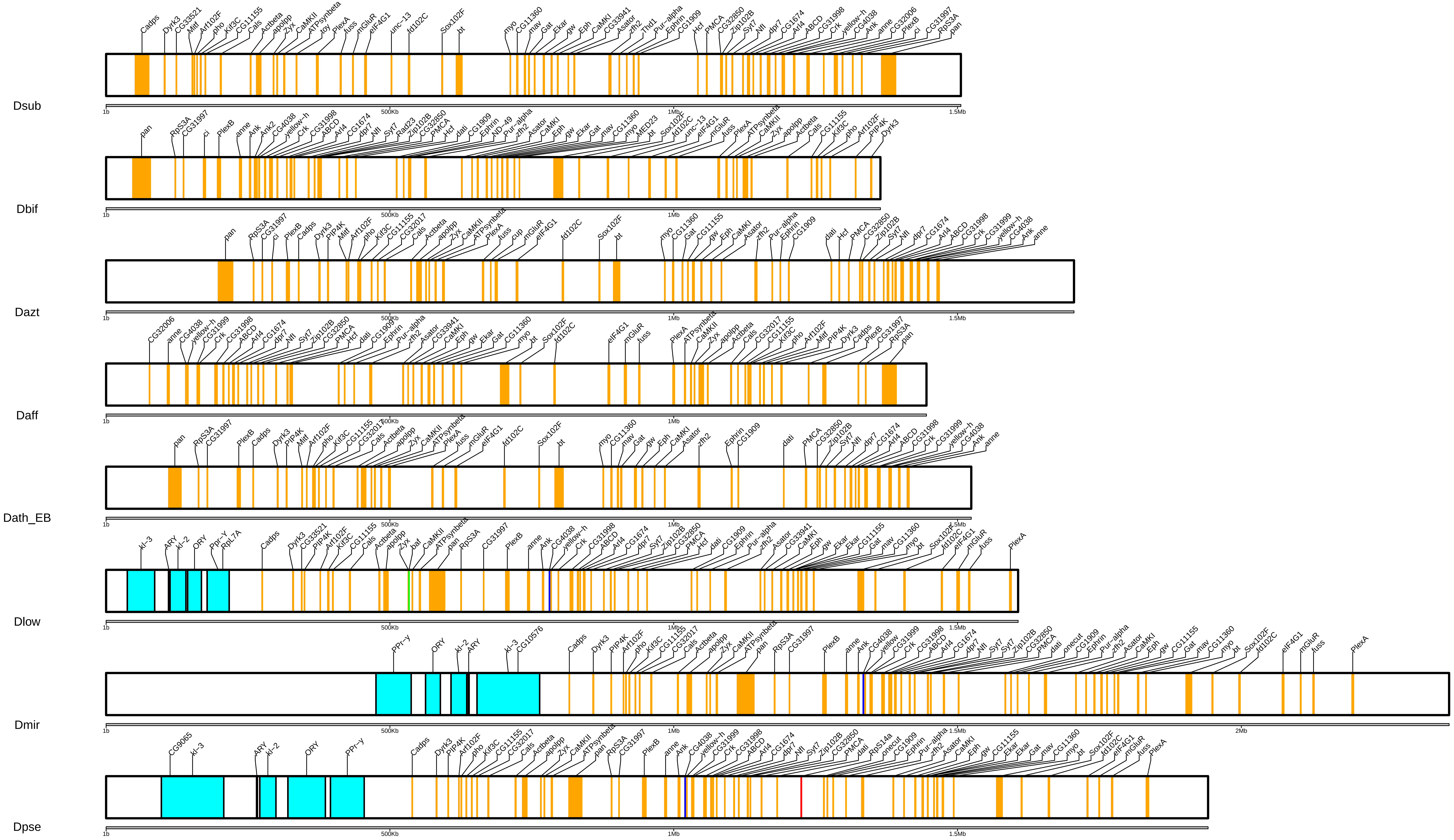

Cadps Dyrk3 CG33521 Mitf Arf102F pho Kif3C CG11155 Cals Actbeta apolpp Zyx CaMKII ATPsynbeta toy PlexA fuss mGluR eIF4G1 unc-13 fd102C Sox102F bt myo CG11360 mav Gat Ekar gw Eph CaMKI CG33941 Asator zfh2 Thd1 Pur-alpha Ephrin CG1909 Hcf PMCA CG32850 Zip102B Syt7 Nfl dpr7 CG1674 Arl4 ABCD CG31998 Crk yellow-h CG4038 Ank anne CG32006 PlexB ci CG31997 RpS3A pan
Dsub
pan RpS3A CG31997 ci PlexB anne Ank Ank2 CG4038 yellow-h Crk CG31998 ABCD Arl4 CG1674 dpr7 Nfl Syt7 Rad23 Zip102B CG32850 PMCA Hcf dati CG1909 Ephrin ND-49 Pur-alpha zfh2 Asator CaMKI Eph gw Ekar Gat mav CG11360 myo MED23 bt Sox102F fd102C unc-13 eIF4G1 mGluR fuss PlexA ATPsynbeta CaMKII Zyx apolpp Actbeta Cals CG11155 Kif3C pho Arf102F PIP4K Dyrk3
Dbif
pan RpS3A CG31997 ci PlexB Cadps Dyrk3 PIP4K Mitf Arf102F pho Kif3C CG11155 CG32017 Cals Actbeta apolpp Zyx CaMKII ATPsynbeta PlexA fuss cup mGluR eIF4G1 fd102C Sox102F bt myo CG11360 Gat CG11155 gw Eph CaMKI Asator zfh2 Pur-alpha Ephrin CG1909 dati Hcf PMCA CG32850 Zip102B Syt7 Nfl dpr7 CG1674 Arl4 ABCD CG31998 Crk CG31999 yellow-h CG4038 Ank anne
Dazt
CG32006 anne CG4038 yellow-h CG31999 Crk CG31998 ABCD Arl4 CG1674 dpr7 Nfl Syt7 Zip102B CG32850 PMCA Hcf dati CG1909 Ephrin Pur-alpha zfh2 Asator CG33941 CaMKI Eph gw Ekar Gat CG11360 myo bt Sox102F fd102C eIF4G1 mGluR fuss PlexA ATPsynbeta CaMKII Zyx apolpp Actbeta Cals CG32017 CG11155 Kif3C pho Arf102F Mitf PIP4K Dyrk3 Cadps PlexB CG31997 RpS3A pan
Daff
pan RpS3A CG31997 PlexB Cadps Dyrk3 PIP4K Mitf Arf102F pho Kif3C CG11155 CG32017 Cals Actbeta apolpp Zyx CaMKII ATPsynbeta PlexA fuss mGluR eIF4G1 fd102C Sox102F bt myo CG11360 mav Gat gw Eph CaMKI Asator zfh2 Ephrin CG1909 dati PMCA CG32850 Zip102B Syt7 Nfl dpr7 CG1674 Arl4 ABCD CG31998 Crk CG31999 yellow-h CG4038 Ank anne
Dath_EB
kl-3 ARY kl-2 ORY PPr-Y RpL7A Cadps Dyrk3 CG33521 PIP4K Arf102F Kif3C CG11155 Cals Actbeta apolpp Zyx baf CaMKII ATPsynbeta pan RpS3A CG31997 PlexB anne Ank CG4038 yellow-h Crk CG31998 ABCD Arl4 CG1674 dpr7 Syt7 Zip102B CG32850 PMCA Hcf dati CG1909 Ephrin Pur-alpha zfh2 Asator CG33941 CaMKI Eph gw Ekar CG11155 Gat mav CG11360 myo bt Sox102F fd102C eIF4G1 mGluR fuss PlexA
Dlow
PPr-y ORY kl-2 ARY kl-3 CG10576 Cadps Dyrk3 PIP4K Arf102F pho Kif3C CG11155 CG32017 Cals Actbeta apolpp Zyx CaMKII ATPsynbeta pan RpS3A CG31997 PlexB anne Ank CG4038 yellow CG31999 Crk CG31998 ABCD Arl4 CG1674 dpr7 Nfl Syt7 Syt7 Zip102B CG32850 PMCA dati onecut CG1909 Ephrin Pur-alpha zfh2 Asator CaMKI Eph gw CG11155 Gat mav CG11360 myo bt Sox102F fd102C eIF4G1 mGluR fuss PlexA
Dmir
CG9065 kl-3 ARY kl-2 ORY PPr-y Cadps Dyrk3 PIP4K Arf102F pho Kif3C CG11155 CG32017 Cals Actbeta apolpp Zyx CaMKII ATPsynbeta pan RpS3A CG31997 PlexB anne Ank CG4038 yellow-h CG31999 Crk CG31998 ABCD Arl4 CG1674 dpr7 Nfl Syt7 Zip102B CG32850 PMCA dati RpS14a onecut CG1909 Ephrin Pur-alpha zfh2 Asator CaMKI Eph gw CG11155 Ekar Ekar Gat mav CG11360 myo bt Sox102F fd102C eIF4G1 mGluR fuss PlexA
Dpse
1b 500Kb 1Mb 1.5Mb 2Mb
